# Protection of Cattle Elicited Using a Bivalent Lumpy Skin Disease Virus-Vectored Recombinant Rift Valley Fever Vaccine

**DOI:** 10.3389/fvets.2020.00256

**Published:** 2020-05-19

**Authors:** David B. Wallace, A. Mather, P. D. Kara, Leeann Naicker, Nobalanda B. Mokoena, A. Pretorius, T. Nefefe, N. Thema, Shawn Babiuk

**Affiliations:** ^1^Vaccines and Diagnostics Development Programme, ARC-Onderstepoort Veterinary Institute, Pretoria, South Africa; ^2^Department Veterinary Tropical Diseases, Faculty of Veterinary Science, University of Pretoria, Pretoria, South Africa; ^3^Onderstepoort Biological Products SOC Ltd., Pretoria, South Africa; ^4^National Centre for Foreign Animal Disease, Canadian Food Inspection Agency, Winnipeg, MB, Canada; ^5^Department of Immunology, University of Manitoba, Winnipeg, MB, Canada

**Keywords:** lumpy skin disease, recombination, vaccine, Rift Valley fever, cattle

## Abstract

Lumpy skin disease and Rift Valley fever are two high-priority livestock diseases which have the potential to spread into previously free regions through animal movement and/or vectors, as well as intentional release by bioterrorists. Since the distribution range of both diseases is similar in Africa, it makes sense to use a bivalent vaccine to control them. This may lead to the more consistent and sustainable use of vaccination against Rift Valley fever through a more cost-effective vaccine. In this study, a recombinant lumpy skin disease virus was constructed in which the thymidine kinase gene was used as the insertion site for the Gn and Gc protective glycoprotein genes of Rift Valley fever virus using homologous recombination. Selection markers, the enhanced green fluorescent protein and *Escherichia coli* guanidine phosphoribosyl transferase (gpt), were used for selection of recombinant virus and in a manner enabling a second recombination event to occur upon removal of the gpt selection-pressure allowing the removal of both marker genes in the final product. This recombinant virus, LSD-RVF.mf, was selected to homogeneity, characterized and evaluated in cattle as a vaccine to show protection against both lumpy skin disease and Rift Valley fever in cattle. The results demonstrate that the LSD-RVF.mf is safe, immunogenic and can protect cattle against both diseases.

## Introduction

Capripoxviruses are large double-stranded DNA viruses in the family Poxviridae comprised of sheeppox, goatpox and lumpy skin disease viruses. Lumpy skin disease (LSD) causes fever and skin lesions in cattle leading to major economic losses as a result of high morbidity, and occasionally mortality (1). No country in sub-Saharan Africa claimed freedom from this disease since its discovery (OIE, HandiSTATUS, 02/02/05). LSD has rapidly spread from its historical range in most of Africa, through the Middle East into Eastern Europe (2) and many countries in Asia (3), currently as far as China, Bangladesh and India. The disease is most likely spread by insect and other arthropod vectors via mechanical transmission and is not highly contagious in the absence of potential vectors. This makes control of LSD difficult and impractical, even in non-endemic countries experiencing occasional incurrences, which have often used stamping-out through slaughter of infected cattle. Effective control can, however, be achieved through vaccination using live attenuated vaccines, as was more recently demonstrated in Europe using mass vaccination (4). Lumpy skin disease and sheep and goat pox are ranked in the top 20 global diseases for impact on the poor (5).

Rift Valley fever virus (RVFV) is an RNA virus and member of the Phenuiviridae family (6) originally described in the 1930's in Kenya in the Rift Valley (7). The geographic distribution of Rift Valley fever (RVF) was limited to sub-Saharan Africa until outbreaks in Egypt in the 1970's (8). In 2000, RVFV spread into Yemen and Saudi Arabia (9). Fortunately, the outbreak was contained and it appears that RVFV is not currently present in the Middle East. However, there is always the risk of new outbreaks, which could become endemic. Rift Valley fever virus is a vector-borne pathogen spread by mosquitos and can infect a wide range of ruminants including cattle, sheep, goats and camels. In livestock, disease is characterized by fever, diarrhea, and abortions. Unlike LSDV, RVFV is a zoonotic virus that regularly causes epidemics among people (10, 11). As a zoonotic disease, RVF is ranked in the top 10 globally. The virus is also of major economic importance in livestock across sub-Saharan Africa and more recently in the Middle East, primarily as a result of trade embargoes on exports of mainly sheep from Africa to the Arabian Peninsula. There are vaccines available for controlling RVF and numerous experimental vaccines in development (12, 13). Since both LSDV and RVFV infect cattle and share the same geographic distribution in Africa, a bivalent vaccine able to protect against both diseases would be advantageous. This can be achieved by either formulating a vaccine combining a live attenuated LSD vaccine strain together with a live attenuated RVF vaccine or by generating a single, recombinant vaccine with protective components of both viruses.

Previously, an LSD-vectored candidate vaccine expressing the RVFV Gn (G2) and Gc (G1) glycoproteins was developed by the Agricultural Research Council-Onderstepoort Veterinary Institute (ARC-OVI) in collaboration with Onderstepoort Biological Products (OBP Ltd). This vaccine utilizes the South African live attenuated Onderstepoort LSD vaccine as vector, which co-expresses the RVFV structural glycoprotein (GP) genes under control of the vaccinia virus P7.5K early/late promoter inserted into the LSDV viral thymidine kinase gene (14). Two selectable marker genes are also present (the *E. coli* guanine phosphoribosyl transferase (gpt) gene and the enhanced green fluorescent protein (EGFP) gene), which assist with the initial stages of recombinant virus selection. This bivalent LSD-RVF vaccine has been shown to fully protect mice and 1-year old Merino sheep against virulent RVFV challenge, in preliminary safety and efficacy tests (15).

However, not only is this bivalent vaccine classified as a genetically modified organism (GMO), but it also retains the dominant selection marker gene (gpt) conferring drug resistance, in addition to the EGFP marker gene. In order to satisfy environmental concerns over the use of products developed from micro-organisms expressing resistance genes and the sharp rise in pathogens in the field acquiring drug resistance (e.g., XDR tuberculosis) (16) it is required that both marker genes are removed in order to satisfy regulatory agencies requirements for licensing the vaccine. This is routinely achieved for other poxvirus-vectored vaccine constructs using transient dominant selection (17, 18). As described in this paper, this method was employed to produce a selection marker-free LSD-RVF candidate vaccine, LSD-RVF.mf. Furthermore, this vaccine requires evaluation in the target species (cattle) to demonstrate its safety and efficacy, as there are numerous examples of vaccines which demonstrated protection in animal models, but failed when used in the target species.

The advantages of using a bivalent LSD-RVF.mf vaccine over conventional vaccination approaches include: (1) a vaccine which is cost effective; (2) since RVF outbreaks are cyclical, producers are reluctant to vaccinate as it is expensive, and thus having a bivalent capripoxvirus vaccine removes these economic reasons for not vaccinating; and (3) the bivalent vaccine is able to differentiate infected from vaccinated animals (DIVA) for RVF, allowing for serological surveillance testing to still be performed when it is in use. The impact of using the bivalent LSD-RVF.mf vaccine includes: (1) preventing mortality and debilitating disease in cattle caused by LSD and RVF in all regions of sub-Saharan Africa where the diseases both occur, leading to improved production and economic development; (2) indirectly protecting people from RVF virus by decreasing the viral loads in livestock; and (3) helping provide an effective barrier to further spread of RVFV into non-endemic countries.

Determining the precise impact of these diseases on incomes of the poor would require a dedicated study. However, bearing in mind the fact that LSD and RVF viruses are OIE listed diseases and the current vaccine uptake of individual vaccines against these diseases is relatively high, this vaccine could become more widely used and have greater benefit in developing countries than most other livestock vaccines currently available. A recent study determined that vaccination against LSD is cost effective and yields a significant investment return to both smallholder and large commercial producers (19).

Since currently used live attenuated LSD and RVF vaccines are effective following a single dose, any new vaccine must also be effective following a single administration in order to be used in the field. Therefore, the LSD-RVF.mf vaccine was evaluated in cattle for its ability to provide protection using a single administration against both virulent LSD and RVF challenge and the results are presented here.

## Materials and Methods

### Cells and Viruses

#### Cells

Primary fetal bovine testes (FBT) cells were prepared according to standard techniques (20). These were used for construction and selection of the recombinants and were propagated in a 1:1 combination of Dulbecco's modified Eagle's medium (DMEM) and Ham's F12 medium (Gibco, USA) containing 8% FCS and antibiotics (100 μg/ml penicillin, 100 μg/ml streptomycin and 250 μg/ml amphotericin B) (Lonza, USA).

Focus selection and titrations were performed using Madin-Darby bovine kidney (MDBK) cells (obtained from the American Type Tissue Culture Collection, no. CCL-22, USA) and baby hamster kidney (BHK) cells (obtained from the American Type Tissue Culture Collection, no. CCL-10, USA) for preparation of the RVFV challenge strain, as below.

#### Viruses

The South African Neethling vaccine strain of LSDV, produced by Onderstepoort Biological Products (OBP LTD), was used as parental virus (21) for the generation of recombinants.

The virulent LSDV Warmbaths field isolate (LSDV_WB) (GenBank Accession no. AF409137) was used as the inoculum strain for the LSDV challenge (22).

The M35/74 field strain of RVFV was used as the inoculum for the RVFV challenge (23). A freeze-dried ampule containing the virus was reconstituted in 1 ml Eagle's Minimum Essential Medium (EMEM) (Gibco, USA) and seeded onto a 90% confluent monolayer of BHK cells in EMEM (with 5% FCS and antibiotics). The infected cells were incubated at 37°C and were freeze-thawed between−20°C and room temperature (RT) three times 2 days after cytopathic effects (CPE) were observed. The lysed cell/virus-containing medium was then aliquoted into 15 ml sterile conical tubes and stored at−20°C, and aliquots removed on two separate occasions for titration on BHK cells. On Day 0 of the trial, the required volume of inoculum was removed from a tube after thawing at RT, followed by mixing, and dilution to the required virus concentration in EMEM.

High-titer virus stocks of the commercial LSD vaccine and the recombinant LSD-RVF.mf vaccine were prepared and obtained from OBP LTD, following their routine manufacturing protocols (details of this procedure is protected intellectual property of the manufacturer).

### Plasmid Design

The insertion vector, pLS(EG)-RV, was constructed along similar lines to pLSEG-RVFV (14), except the transfer vector, pLS(EG), was developed with insertion of the positive and visual selection marker genes, the *E. coli* gpt and EGFP genes respectively, exterior to the TK-L and TK-R LSDV thymidine kinase (TK) gene flanking regions (Supplementary Material 1).

### Recombinant Virus Construction, Selection and Characterization

The recombinant marker-free virus was generated, selected and characterized according to the improved method described by Wallace et al. (24), with the addition of a second round of selection in the absence of mycophenolic acid (MPA) selection pressure, resulting in a product free from both marker genes [known as transient dominant selection (17, 18)]. However, as a potential by-product of this selection method is a reconstituted parental virus (with an intact TK gene and no RVFV GP genes), a method was devised to enable selection of homogeneous marker-free recombinant virus expressing the RVFV GPs–in effect, an “intracellular” ELISA, as described below.

### “Intracellular” ELISA

Ninety-six well cell culture plates (Nunclon, Denmark) were seeded with FBT cells and once 80–90% confluent, growth medium was removed, replaced with maintenance medium and infected with half aliquots of pre-selected foci (the remainder of each focus being frozen at −20°C) in duplicate (uninfected cells and parental LSDV-infected cells were included as controls).

Once the cells were heavily infected (displaying >80% cytopathogenic effect [cpe]), they were washed gently in PBS, fixed with ice-cold 70% acetone for 10 min (min) and allowed to dry overnight at 4°C. The fixed cells were then incubated at 37°C for 1 h in 10% fat-free milk powder (Elite, Clover, South Africa) (diluted in PBS) (300 μl/well), washed three times in Tris-buffered saline (with Tween 20, pH 8.0) (TST) wash buffer (Sigma, USA) (300 μl/well) and then incubated at 37°C for 1 h with 100 μl/well positive RVFV polyclonal sheep serum diluted 1:50 in 10% fat-free milk powder. The washing was repeated (as above) and the cells incubated for 1 h at 37°C with 100 μl/well Protein G-conjugated horseradish immunoperoxidase (Invitrogen, USA) diluted 1:10 000 in 10% fat-free milk powder. After another round of washing, 100 μl/well of TMB Ready-to-use substrate (Life Technologies [ThermoFisher Scientific, USA]) was added for 10 min and thereafter the color reaction development was stopped using sulphuric acid (Merck, USA) and the OD values were determined using a BioTek ELISA reader (Model: ELX808) (BioTek, USA) at 450 nm.

OD values for each duplicate set of wells were averaged and using the negative control well values as a guide, wells with the highest average OD values were selected as those containing recombinant virus at or close to homogeneity and expressing high levels of the RVFV GPs.

Frozen half-aliquots of foci, selected due to their high OD values, as above, were removed from −20°C storage, thawed and frozen three times, and added to fresh uninfected FBT cells. Released virus was propagated and once cells displayed 100% cpe, viral and cellular DNA was purified using the Roche Magna Pure Total nucleic acid extraction kit (Roche, Switzerland) and a Roche MagNA Pure LC 2.0 extractor according to the manufacturer's instructions. Viral foci were then characterized for homogeneity (free from parental LSDV virus and EGFP and gpt marker genes) using PCR-amplification of the target insertion region, as described next.

### Polymerase Chain Reaction (PCR) Assay

Detection of recombinant viruses containing the RVFV GP genes and selection to homogeneity was performed using conventional PCR. In brief, a primer pair (MP1-F: 5′ – CTC CTG TAT TTA TAG AAC CTA – 3′; MP1-R: 5′ – GCA TTA TCA TTA TCG TCA TCA TC – 3′) was designed to amplify flanking regions of the LSDV TK gene (product size ~ 1.9 kilo-base pairs [kbp]), into which the RVFV GP genes were inserted (Supplementary Material 1). Amplification was carried out using a GeneAmp 2400 thermocycler (Perkin-Elmer, USA) in a 25 μl reaction volume consisting of 2.5 μl 10X PCR buffer (containing 20 mM MgCl_2_), 2 μl 2.5 mM dNTPs, 0.25 U Taq DNA polymerase (TaKaRa Ex Taq^TM^) (Takara Biomedicals, Japan), 0.5 μl of each primer [20 pmoles each] [Gibco-BRL, UK], 1 μl template DNA (~ 0.1 ng) and 18.4 μl sterile distilled water. Template DNA was denatured for 45 s (sec) at 95°C, primer annealing was carried out at 62°C for 45 sec, and strand extension was at 72°C for 7 min (repeated through 35 amplification cycles) (preceded by an initial denaturation step at 95°C for 60 s and a final elongation step at 72°C for 7 min and then holding at 4°C). PCR products were separated on a 2% agarose gel containing Ethidium bromide (EtBr).

### Indirect Immunofluorescence Assay (IFA)

The IFA was used to detect the expression of the RVFV glycoprotein genes from the recombinant virus using the method described by Wallace (25), The slides were viewed and images captured using an Olympus BX41 phase contrast microscope (Olympus, USA) and an Olympus DX10 digital camera (Olympus, Japan).

### Animal Selection, Care, and Housing

The cattle trial was conducted with the approval of the Agricultural Research Council-Onderstepoort Veterinary Institute Animal Ethics Committee (approval no. AEC 4.17) and the South African Department of Agriculture, Forestry and Fisheries (DAFF) (Section 20 of the South African Animal Diseases Act [Act No 35 of 1984] permit no. 12/11/1/1/15).

Fifty-six male Holstein-Friesian cross calves, ~8 months old, were pre-screened for exposure/presence of LSDV and RVFV using a conventional PCR for LSDV (26) and a real-time RT-PCR for RVFV (27), respectively. Serology was also performed for detecting antibodies to either pathogen. Forty calves were then selected, purchased and delivered to the ARC–Transboundary Animal Diseases (TADs) biosafety containment level-3 facilities, of which twenty were used in this trial.

They were housed in four individual stalls with five animals per stall. During a 2-weeks acclimatization period, the calves were monitored for overall health and well-being. Rectal temperatures were recorded throughout this period and throughout the trial. The calves were fed daily on a balanced maintenance diet of bovine pellets and provided with fresh, clean water *ad-libitum*. Within the 1st week of arrival, serum samples were obtained from each calf and retested for the presence of LSDV and RVFV antibodies, as described. For blood collection, animals were first mildly sedated with Xylazine-hydrochloride (Rompun^Tm^, Bayer AH, USA) and once calm they were bled via the jugular vein into EDTA or serum tubes. At the conclusion of the trial, all calves were euthanised using an overdose of sodium pentobarbitone (Sigma-Aldrich, USA).

### Vaccination and LSD Challenge

The calves were inoculated as described in Table 1. The 10 calves in Group 1 were mock-vaccinated with 2.0 ml vaccine diluent subcutaneously (SC) (obtained from OBP Ltd, South Africa) as negative controls, while calves in Group 2 were inoculated SC with 2.0 ml of the LSD-RVF.mf construct at a titer of 1.0 x 10^3^ TCID_50_/ml.

**Table 1:** Calf identity numbers, group numbers, the inoculum used for both inoculation and challenge and their relevant titres and routes and day of challenge.

**Animal number**	**Group**	**Inoculum (titer)**	**Day of challenge and challenge virus**	**Titer**
D1-1	1	diluent^*^ (2.0 ml, SC)	Day 21 post-inoculation. LSDV Warmbaths isolate	5 × 10^4^ TCID_50_/ml (2.0 ml, SC) & 5 × 10^5^ TCID_50_/ml (2.0 ml, IV)
D1-2				
D1-3				
D1-4				
D1-5				
D2-1		diluent^*^ (2.0 ml, SC)	Day 28 post-inoculation. RVFV strain M35/74	3.0 × 10^6^ TCID_50_, 1.0 ml IV
D2-2				
D2-3				
D2-4				
D2-5				
A1-1	2	LSD-RVF.mf (1,0 × 10^3^ TCID_50_)	Day 21 post-inoculation. LSDV Warmbaths isolate	5 × 10^4^ TCID_50_/ml (2.0 ml, SC) & 5 × 10^5^ TCID_50_/ml (2.0 ml, IV)
A1-2				
A1-3				
A1-4				
A1-5				
C2-1		LSD-RVF.mf (1,0 × 10^3^ TCID_50_)	Day 28 post-inoculation. RVFV strain M35/74	3.0 × 10^6^ TCID_50_, 1.0 ml, IV
C2-2				
C2-3				
C2-4				
C2-5				

* *2.0 ml of vaccine diluent, obtained from Onderstepoort Biological products*.

At 21 day's post-inoculation (dpi), five of the mock-vaccinated calves in Group 1 (D1-1 to D1-5) and five of the calves vaccinated with the LSD-RVF.mf construct in Group 2 (A1-1 to A1-5) were challenged with 2.0 ml of LSDV_WB at a titer of 5.0 × 10^4^ TCID_50_/ml, clustered at two sites SC, and 2.0 ml intravenously (IV) at a titer of 5.0 × 10^5^ TCID_50_/ml.

#### Detection of LSDV Viremia

Whole blood was collected from calves on the day of inoculation and 7 and 14 dpi, and similarly, equivalent days' post-challenge (dpc). The blood was aliquoted into sterile 1.5 ml tubes (Eppendorf, Germany). An equal volume of Lysis/binding buffer from the MagNA Pure LC total nucleic acid isolation kit (Roche, Switzerland) was added to the blood, which was then frozen at −20°C until further use. Once the samples were thawed, total nucleic acid extraction was performed using the kit (following its “Total NA HP” protocol) and a Roche MagNA Pure LC 2.0 extractor, both according to the manufacturer's instructions.

Detection of the extracted LSDV DNA was achieved using a conventional PCR and a GeneAmp 2400 thermocycler (Perkin Elmer, USA). The method was performed as described by Viljoen et al. (26). The primer pair (OP3: 5′-CAC CAG AGC CGA TAA C−3′; OP49: 5′-GTG CTA TCT AGT GCA GCT AT−3′) was used. This primer pair amplifies a 450 base pair (bp) region within the wild type LSDV TK gene. Amplification was carried out using a GeneAmp 2400 thermocycler (Perkin-Elmer, USA) in a 25 μl reaction volume consisting of 2.0 μl 10X HotStarTaq PCR buffer, 1 μl 2.5 mM dNTPs, 2 units HotStarTaq polymerase (Qiagen, Germany), 1.0 μl of each primer [20 pmoles each] [Gibco-BRL, UK], 5.0 μl template DNA (1 ng) and 14.8 μl sterile DNase/RNase free water. Template DNA was denatured for 45 s (sec) at 94°C, primer annealing was carried out at 53°C for 45 s, and strand extension was at 75°C for 60 s (repeated through 35 amplification cycles) (preceded by an initial denaturation step at 95°C for 7 min). PCR products were separated on a 2% agarose gel containing Ethidium bromide (EtBr).

#### Detection of LSDV in Skin Lesions

Biopsies of skin lesions were collected from the calves as described by Tuppurainen et al. (28), with some modifications: instead of suturing the wound, the site was sprayed with antiseptic spray (Zeropar, Bayer AH, USA) daily for 3 days following the procedure. Prior to the biopsy, the area was first sterilized using a cotton swab soaked in 70% ethanol. Skin lesion samples were sent for immunohistopathology evaluation specific for detection of LSDV antigen and gross cellular pathology related to virus infection (Idexx Laboratories, South Africa).

#### Humoral Immunity

Sera was recovered from blood collected from all calves on the day of inoculation, and 7, 11, 17, and 21 dpi, the day of challenge and 8, 11, 14, and 21 dpc. Detection of antibodies in sera to LSDV was achieved using the virus neutralization test (VNT) and the constant virus-varying serum method (29). For RVFV antibody detection, the standard VNT (30) was used and two ELISAs–a RVFV immunoglobulin M (IgM) capture ELISA (31) and an immunoglobulin G (IgG) indirect ELISA (32).

#### Cellular Immunity

Whole blood was collected on the day of inoculation, 7 and 14 dpi, the day of challenge and 7 and 14 dpc. Lymphocytes were isolated and used for bovine IFN-γ ELISPOT and phenotype analysis as described previously by Kara et al. (22). In both assays PMBCs (2 × 10^5^ cells/well) were stimulated in triplicate with the LSDV_WB isolate (1 × 10^5^ pfu/well), inactivated whole RVFV antigen (0.5% [V/V] formaldehyde solution) (10 μg/ml, from OBP LTD, Smithburn vaccine strain) and positive ConA antigen (5 μg/ml, Sigma Aldrich, USA). Responses were measured using the bovine IFN-γ ELISPOT kit (Mabtech AB, Sweden) as per the manufacturer's instructions and spot-forming cells (SFC) were enumerated using an automated ELISPOT reader (Zeiss KS ELISPOT Compact 4.5, Germany). Antibodies used for phenotype determination (at a 1:100 dilution) were mouse anti-bovine CD45Ro-PE (cell line IL-A116, IgG3, BioRad), mouse anti-bovine CD8α-FITC (Clone 38.65, IgG1, BioRad), mouse anti-bovine CD4 primary antibody (cell line GC50A1, IgM, VMRD Inc, USA) and goat anti-mouse IgM-APC secondary antibody (1:8 dilution; Invitrogen, UK). Flow cytometry data acquisition was performed using a MACSQuant® Analyzer 10 (Miltenyi Biotec) and data were analyzed using Kaluza 2.1 software (Beckman Coulter, USA). Isotypic controls were included for all isotypes used. The significance of differences between the average values per group was determined by means of the Student's *t*-test. Differences with *P* ≤ 0.05 were considered significant.

### RVF Vaccination and Challenge

Twenty-eight dpi the remaining five mock-vaccinated calves in Group 1 (D2-1 to D2-5) and the five calves inoculated with the LSD-RVF.mf construct in Group 2 (C2-1 to C2-5) were challenged intravenously with the virulent RVFV M35/74 strain at a titer of 3.0 × 10^6^ TCID_50_/ml in 1.0 ml (Table 1).

Sera were recovered from blood collected from all calves on the day of inoculation, and 7, 11, 17, and 21 dpi, the day of challenge and 3, 6, 9, and 14 dpc, and were evaluated for RVFV-specific antibodies. Whole blood was collected pre-challenge for CMI testing, as described.

#### Detection of RVF Viremia

A RVFV real-time RT-PCR (27) was used for the detection of RVFV in serum samples that were recovered on the day of challenge and 3, 6, 9, 14, and 21 dpc.

Total nucleic acid was extracted from serum samples using the MagNA Pure Total Nucleic Acid Kit (High Performance) (Roche, Switzerland). The real-time RT-PCR assay was performed using the LC 480 One-step RNA Master kit (Roche, Switzerland), as per the manufacturer's instructions. The following primers and probe were used: RVS (Forward): 5′- AAA GGA ACA ATG GAC TCT GGT CA−3′; RVA (Reverse): 5′- CAC TTC TTA CTA CCA TGT CCT CCA AT−3′; RVP (Probe): 5′ –FAM- AAA GCT TTG ATA TCT CTC AGT GCC CCA A –BHQ−3′. The real-time RT-PCR reaction mix and the cycling conditions are listed in Supplementary Material 2.

## Results

### Recombinant Virus Construction, Selection and Characterization

The OBP vaccine strain of LSDV was used as the parental virus with the pLS(EG)-RV insertion plasmid containing the RVFV GP genes and EGFP and *E*. *coli* gpt selection marker genes to generate recombinant virus through the use of homologous recombination (Supplementary Material 1) (24). Selection of foci with EGFP expression under mycophenolic acid (MPA) selection pressure was achieved through multiple rounds of plaque picking, followed by the progeny virions being grown in the absence of MPA to allow for removal of the selection markers. EGFP fluorescence was rapidly lost from these foci, indicating occurrence of a second recombination event resulting in either reversion to parental virus, or recombinant viruses containing the RVFV GP genes, but with the loss of the EGFP and gpt selection marker genes - LSD-RVF.mf.

Multiple foci lacking EGFP expression were selected, half portions of each were frozen at −20°C and the remainder subjected to the “Intracellular ELISA” technique developed and described under the Materials and Methods. Wells containing foci showing signals (OD values) for the positive RVFV antiserum which were significantly higher than in duplicate wells for the negative serum were deemed to be positive for expression of the RVFV GPs and were further selected and sub-cultured after filtration and sonication (Supplementary Material 3). DNA was extracted from a number of sub-cultured foci resulting from focus 2a.2 and PCR was performed to confirm homogeneity (Supplementary Material 4). The presence of amplification products of the correct size (~ 5.5 kpb) for the recombinant virus and absence of any amplification products of 1.9 kbp, indicative of parental virus, provided clear evidence that the sub-cultures were pure and thus free of parental virus.

**Figure 1:**
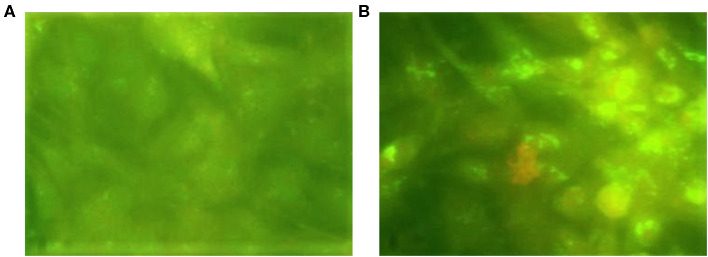
LSD-RVF.mf recombinant foci immunostaining of **(A)** Negative control–an unrelated LSDV-vectored recombinant construct (expressing an antigen of Mycoplasma mycoides subspecies mycoides small colony) and **(B)** Sub-culture foci 2a.2 (x100).

Recombinant virus foci were also confirmed for expression of the RVFV GPs using the IFA, as shown in Figure 1. Only the LSD-RVF.mf foci were positive for RVFV protein expression.

Sub-culture focus 2a.2_6 was finally selected for a stability study, in which it was passaged 10x in cell culture and retested for RVFV GP expression and homogeneity (data not shown), and it was shown to be stable, homogeneous and expressing the RVFV GPs. This recombinant virus was then grown to high titres, aliquots made and stored at −20°C, until further use.

### Vaccine Safety in Cattle

All animals tested negative for the presence of either LSDV or RVFV by the methods used for pre-screening, and were deemed to be in good health at the outset of the trial.

**Figure 2:**
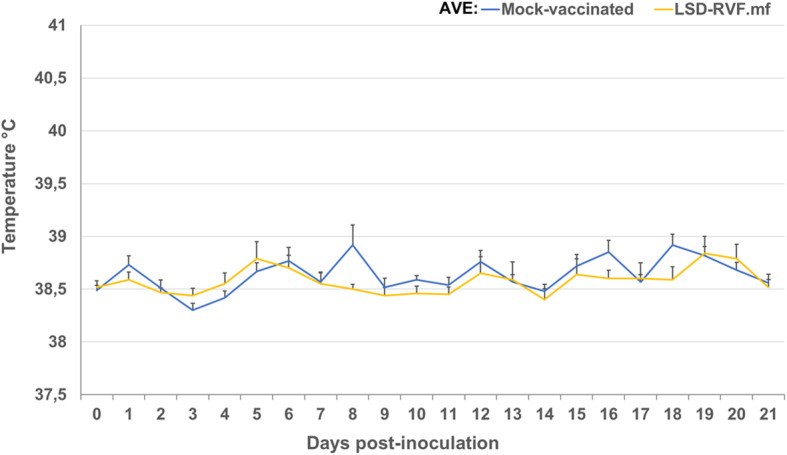
Average temperatures of calves following vaccination with LSD-RVF.mf vaccine and mock vaccination with PBS. No increased rectal temperatures indicative of pyrexia (>39.5°C) were observed for any of the calves.

No erythema or abscesses were observed in any of the negative control animals, post-inoculation. Two of the 10 animals in the vaccinated group (with LSD-RVF.mf) (animals A1-3 and C2-1) developed a mild local reaction at the site of inoculation at 7 dpi. In animal A1-3, this presented as a swelling (2 × 3 cm in size), which persisted to 15 dpi, after which it began to resolve and by 26 dpi it was completely resolved. Animal C2-1 presented with a single injection-site swelling, 1.5 × 1.5 cm in size. A biopsy sample from this region was not positive for LSDV antigen via immunohistochemistry (IHC) staining. No pyrexia (temperature > 39.5°C) was observed in any of the vaccinated animals (Figure 2), nor was it possible to detect LSDV nucleic acid in blood via conventional PCR from the vaccinated group of animals at 7 or 11 dpi.

### Protection Against LSDV Challenge

#### LSD Clinical Evaluation

**Figure 3:**
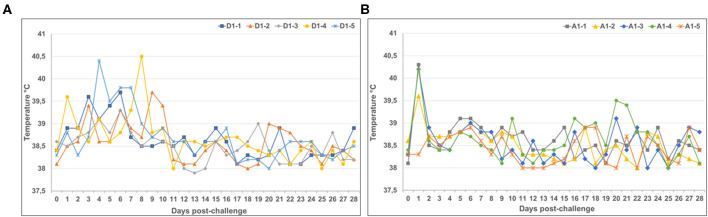
**(A)** Temperatures of mock-vaccinated calves following challenge with the virulent LSDV_WB isolate. Increases in rectal temperatures indicative of pyrexia (>39.5°C) were observed in calf D1-4 and D1-5 at 8 and 4 dpc, respectively. Febrile responses lasted for 4 days in animal D1-5, while the temperature normalized the following day in animal D1-4. **(B)** Temperatures of calves vaccinated with LSD-RVF.mf following challenge with the virulent LSDV_WB isolate. Increases in rectal temperatures indicative of pyrexia were observed in all calves, except animal A1-5, at 1 dpc. Febrile responses (in the range of 39.6–40.3°C) lasted for a single day, with temperatures normalizing by the following day.

Following virus challenge with the virulent LSDV_WB isolate, an increase in rectal temperatures indicative of pyrexia (>39.5°C) was observed in 4/5 of the mock-vaccinated negative control calves (Figure 3A). Animal D1-4 and D1-5, at 8 dpc and 4 dpc, respectively, had temperatures above 40°C. The febrile response lasted for 4 days in animal D1-5, while the temperature normalized the following day in animal D1-4. In the calves vaccinated with LSD-RVF.mf (Figure 3B) an increase in rectal temperatures indicative of pyrexia was observed in all calves, except animal A1-5, at 24 h post-challenge. The febrile response (in the range of 39.6–40.3°C) lasted for a single day with temperatures normalizing the following day.

#### LSD Viremia

Viremia was assessed using detection of LSDV DNA in blood using conventional PCR. Viral nucleic acid was not detected in the control or the LSD-RVF.mf-vaccinated group via conventional PCR at any time points tested, post-challenge.

#### LSDV-Specific Antibody Responses Following Vaccination and Challenge

**Figure 4:**
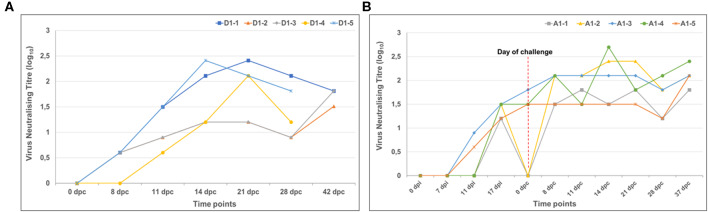
LSDV-neutralizing antibody titres in calves. **(A)** Mock-vaccinated animals had no detectable virus neutralizing antibody levels at any days' post-inoculation (dpi) and are therefore not represented on the graph. However, neutralizing antibody titres were observed in all animals by 11 days' post challenge (dpc) with the highest levels observed at 14 and 21 dpc. **(B)** All calves inoculated with the LSD-RVF.mf vaccine sero-converted by 17 dpi with two animals sero-converting as early as 11 dpi. A significant increase in titres were observed following challenge in three animals by 14 dpc.

Sera were evaluated at multiple time points following vaccination and challenge. No measurable neutralizing antibodies were detected in the mock-vaccinated group after inoculation, however, neutralizing antibody titres were observed in all five animals by 11 dpc, with the highest levels observed at 21 dpc (Figure 4A). Following vaccination with LSD-RVF.mf, LSDV-specific virus neutralizing antibodies were detected as early as 11 dpi in animals A1-2 and A1-5 (Figure 4B). All five animals sero-converted by 17 dpi. A significant increase in titres were observed following challenge in three animals by 14 dpc.

#### LSDV- and RVFV-Specific Cellular Immune Responses

**Figure 5:**
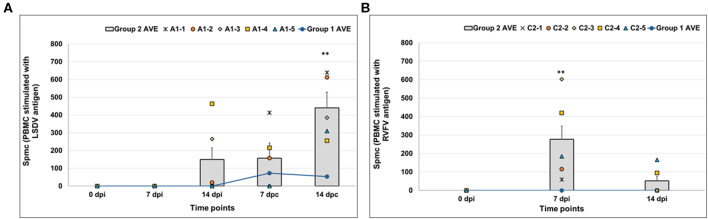
Interferon-γ (IFN-γ) responses in PBMCs collected from calves. **(A)** Group 2 (vaccinated and LSDV challenged) animals' PBMCs stimulated with LSDV. **(B)** Group 2 (vaccinated and RVFV challenged) animals stimulated with Rift Valley fever virus (RVFV) antigen. Averages for the Group 1 (mock-vaccinated control animals: D1-1 to D1-5) are depicted in the line graph in both panels. PBMCs were isolated for IFN- γ responses on the day of inoculation (Day 0), 7 and 14 dpi, and 7 and 14 dpc. The average spots per million cells (spmc) for 5 calves are represented for ELISPOT as a bar graph while individual animals' data are also indicated at each time point. Significant differences, as compared to the average 0 dpi data with *P* ≤ 0.01, are indicated with**.

**Figure 6:**
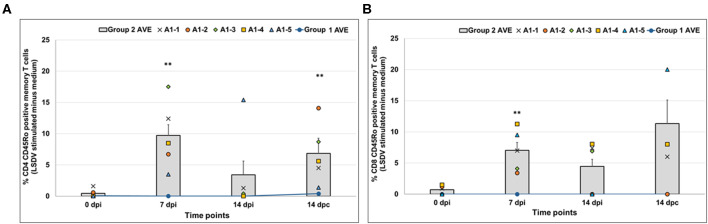
Phenotypic analysis of memory T cells in PBMCs collected from calves. **(A)** CD4+ CD45RO+ cells and **(B)** CD8+ CD45RO+ cells in Group 2 (vaccinated and LSDV challenged animals) when PBMCs were stimulated with LSDV. Averages for the Group 1 (mock-vaccinated control animals: D1-1 to D1-5) are depicted in the line graph in both panels. PBMCs stimulated with antigen were normalized against unstimulated PBMCs. Significant differences, as compared to the negative control group with *P* ≤ 0.01, are indicated with**.

PBMCs were collected from calves on the day of inoculation (day 0), 7 and 14 dpi, and 7 and 14 dpc (Figure 5). ELISPOT analyses showed increased levels of IFN-γ-producing cells at 14 dpi in two animals (A1-3 and A1-4) from Group 2 (the vaccinated and LSDV-challenged group: animals A1-1 to A1-5) when stimulated with LSDV antigen (Figure 5A). Increased numbers of IFN-γ -producing cells were detected in Group 2 calves post-challenge, with higher expression levels in the vaccinated group being recorded (Figure 5A). Numbers of IFN-γ-producing cells were significantly increased at 7 dpi when stimulated with RVFV antigen (Figure 5B) in Group 2 animals (vaccinated and RVFV challenged animals: C2-1 to C2-5). Animals in Group 1 (mock–vaccinated: D1-1 to D1-5) challenged with LSDV also showed an increase in IFN-γ -producing cells post-challenge when stimulated with LSDV antigen (Figure 5A, line graph), while IFN-γ secretion was absent in the PBMCs of the same animals after stimulation with RVFV antigen. The vaccinated group challenged with virulent RVFV (Group 2: C2-1 to C2-5) was not immunologically monitored after challenge due to safety risks. Phenotypic analysis to determine memory T cells indicated that animals in Group 2 (vaccinated and LSDV-challenged animals: A1-1 to A1-5) had a significant increase in both CD4+ CD45Ro+ and CD8+ CD45Ro+ memory T cells at 7 dpi and 14 dpc after stimulation with LSDV antigen (Figures 6A,B). PBMCs stimulated with RVFV antigen only showed an increase in CD8+ CD45Ro+ memory T cells at 14 dpi, while no CD4+ memory T cells were detected (Supplementary Material 5). No memory cells specific to LSDV or RVFV antigen were detected in Group 1 (mock-vaccinated animals: D1-1 to D1-5), as indicated in Figures 6A,B, line graph.

### Protection Against RVF Challenge

#### RVF Clinical Evaluation

**Figure 7:**
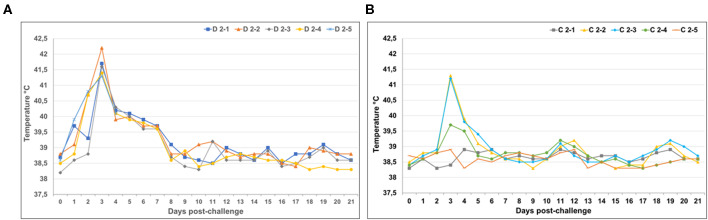
Temperature responses in vaccinated and mock-vaccinated animals following virulent RVFV challenge. **(A)** Rectal temperatures of individual animals in the control mock-vaccinated group. **(B)** Rectal temperatures of individual animals in the vaccinated group.

Animals in the control (mock-vaccinated) group and the vaccinated group responded with pyrexia following challenge, although the mean rectal temperatures were higher in the control group, with peak temperatures occurring in all animals 3 days' post-challenge (dpc) followed by a decline back to baseline (Figure 7A). Animals C2-2 and C2-3 in the vaccinated group developed fevers above 41°C at 3 dpc (Figure 7B).

All animals in the control group displayed typical clinical signs of RVFV infection, such as inappetence and occasional sternal recumbency. Control animal D2-2's temperature peaked at 42.2°C and it developed diarrhea 6 dpc. One control animal (D2-5) died at 5 dpc and was immediately necropsied. The carcass was bloated and partially autolyzed. The liver had multiple coalescing pale areas of necrosis surrounded by hemorrhagic hepatic parenchyma. Petechiae of the mucosa of the gall bladder and diffuse hemorrhaging of lymph nodes were observed – thus, the cause of death was linked to acute RVFV infection. Liver, kidney and spleen samples were sent for histopathology and IHC staining and evaluation related to RVFV infection (Idexx Laboratories, South Africa). Positive staining for RVFV antigen was detected in pooled organ samples from this animal, further supporting cause of death linked to RVFV infection.

None of the animals in the vaccinated group showed clinical symptoms, besides pyrexia, typical of RVFV infection. No gross pathology consistent with RVFV infection was observed in organs of the remainder of the animals in the control group or in any of the animals in the vaccinated group, upon necropsy following euthanasia.

#### RVF Viremia

**Figure 8:**
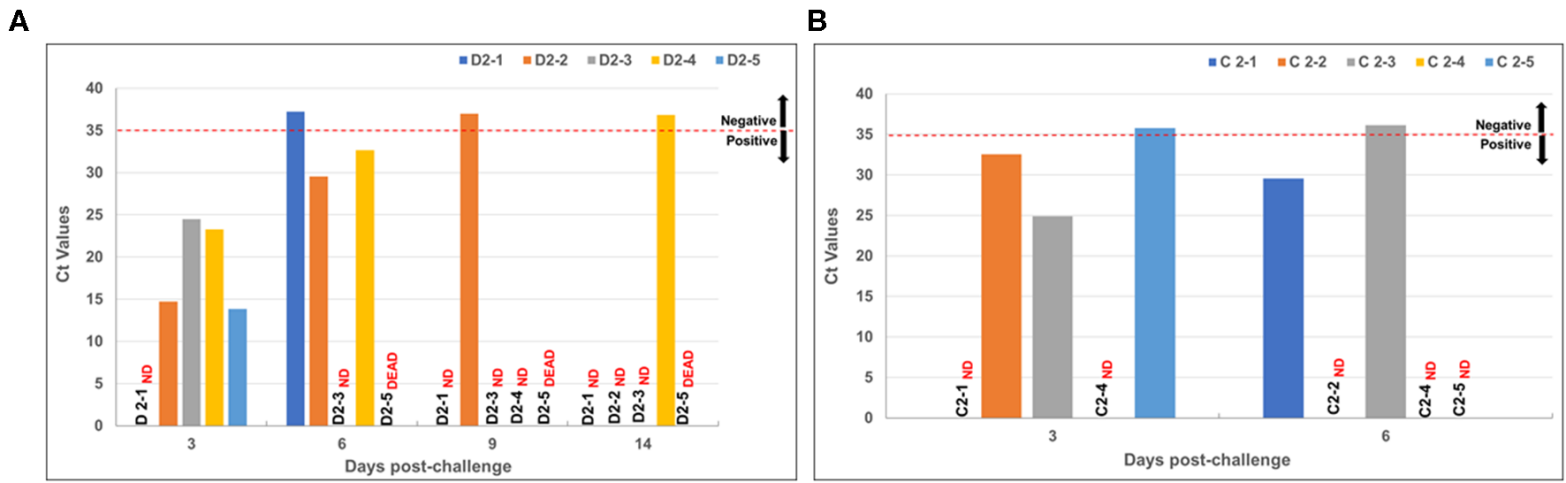
Viremia in vaccinated and mock-vaccinated calves following virulent RVFV challenge. *ND*, not detected and indicates the animal number. Dashed line (- - - -) denotes cut-off point between positive (below line) and negative (above line). **(A)** - qRT-PCR Ct values for mock-vaccinated animals and **(B)** vaccinated animals after challenge with RVFV.

Viremia was assessed by detecting RVFV genomic sequences in sera using qRT-PCR. Viral genomes were detected in the mock-vaccinated calves 3 dpc in four of the five animals (not detected in animal D2-1) (Figure 8A). Peak levels of RVFV occurred at this time point for most animals as indicated by low Ct values. Animal D2-5 displayed the lowest Ct value at 3 dpc, and succumbed to RVFV infection by 5 dpc. In contrast, RVFV was detected in two of the five animals (C2-2 and C2-3) in the vaccinated group and displayed higher Ct values than the mock-vaccinated group at 3 dpc (Figure 8B). By 6 dpc, RVFV was detected in only one animal (C2-1) at a low level, as indicated by the high Ct value.

#### RVFV-Specific Antibody Responses Following Vaccination and Challenge

**Figure 9:**
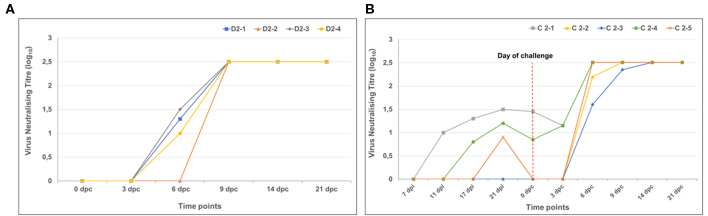
RVFV neutralizing antibody titres in calves. **(A)** Mock-vaccinated animals had no detectable virus neutralizing antibody levels at any days' post-inoculation (dpi) and are therefore not represented on the graph. Highest levels of neutralizing antibody titres were observed in all animals by 9 dpc. Animal D2-5 is not shown on the graph as neutralizing antibodies were not detected at 3 day's post-challenge (dpc), however, the animal died of acute RVF at 5 dpc. **(B)** Three calves inoculated with the LSD-RVF.mf vaccine sero-converted post-vaccination. A significant increase in titres were observed following challenge in all animals by 9 dpc (testing was performed up to a limit of 1:320 serum dilution, thus all final titres are higher).

The mock-vaccinated animals had no detectable virus neutralizing antibody levels post-inoculation. Virus neutralizing titres post-challenge were higher in the vaccinated group than in the mock-vaccinated group by 6 dpc (Figure 9A). Rift Valley fever virus neutralizing antibodies were present in three of the five calves (Figure 9B) after vaccination, which increased significantly after challenge by 6 dpc.

**Figure 10:**
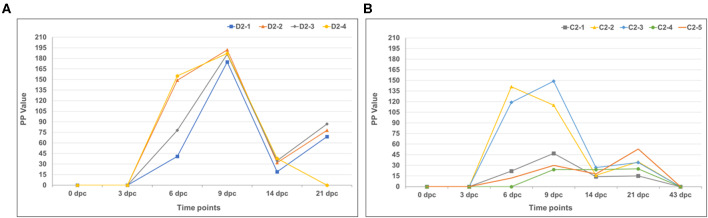
Immunoglobulin M (IgM) host antibody responses demonstrated using a RVFV-specific recombinant nucleoprotein (rNP) capture enzyme-linked immunosorbent assay (ELISA). **(A)** Mock-vaccinated calves and **(B)** LSD-RVF.mf-vaccinated calves.

**Figure 11:**
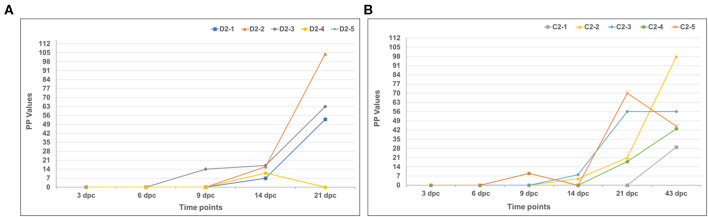
Immunoglobulin G (IgG) host antibody responses demonstrated using a RVFV-specific recombinant nucleoprotein (rNP) indirect enzyme-linked immunosorbent assay (ELISA). **(A)** Mock-vaccinated calves and **(B)** LSD-RVF.mf-vaccinated calves.

The sera were also evaluated for antibodies to RVFV using two ELISAs (an IgG and IgM ELISA) utilizing recombinant RVFV nucleoprotein (rNP) as antigen (Figures 10, 11). As expected, first IgM antibodies were detected, from 3 dpc, and then IgG antibodies, from 6 dpc, since the recombinant LSDV-vectored RVF vaccine expresses the viral GPs, and not the NP. IgM antibody levels rapidly decreased from 9 to 14 dpc, then rose around 21 dpc. The IgG antibody levels (Figure 11) rapidly rose between 14 and 21 dpc for both mock-vaccinated and vaccinated groups, although not for all animals.

## Discussion

Lumpy skin disease is no longer an “African” disease, as it has spread into the Middle East, Eastern Europe, the Balkans, Russia and Kazakhstan, with rapid recent spread into China, Bangladesh and India, which significantly increases the risk of LSD spreading into the remaining free countries in Asia. Currently, only the Americas, Western Europe, Australia, New Zealand, Indo-Pacific islands, amongst others, can claim to be LSD-free. However, these regions face a constant threat of inadvertent or intentional introduction.

In contrast, so far Rift Valley fever has remained within Africa, except for infrequent spread to Saudi Arabia and Yemen in the Middle East. It is possible that Rift Valley fever virus could spread into new regions, including the Americas, as was the case with West Nile virus. Increasing socio-political unrest and bioterrorism have drastically increased the risk for further spread of the disease.

Vaccination is the proven method for controlling both human and animal diseases, and its use has been demonstrated to successfully control LSD and RVF. Although efficacious vaccines exist for both, due to the sporadic nature of RVF outbreaks requiring factors such as weather and mosquito vectors acting together to produce a “perfect storm,” vaccination against the disease is not practiced consistently. This results in increasing the susceptible ruminant population over time. Outbreaks of LSD, by contrast, generally occur annually, therefore vaccination is practiced more regularly. For many smallholder farmers across Africa, the high cost of vaccines, lack of education on their many benefits, and limited access and available infrastructure to enable their proper storage at low temperatures, are some factors which impede their regular use. In addition, not all governments provide aid to support vaccination programs. The development and use of multivalent and combination vaccines in the medical and veterinary field have offered a partial solution to these problems. Lumpy skin disease virus has been investigated for its ability to perform as a stable and versatile vaccine delivery platform, as has been the case for other poxviruses such as vaccinia virus and canarypox virus, but with the added advantage of providing additional protection against LSD – and, sheep- and goat-pox (12). Since vaccination for LSD is routinely practiced, having a bivalent- or multivalent-vectored vaccine based on LSDV has advantages compared to conventional vaccination using multiple vaccines.

In the past, we developed and evaluated recombinant LSDV-vectored vaccines, including one expressing the protective glycoproteins (GPs) of RVFV, with retention of the selectable markers used for their generation and selection to homogeneity (15). However, due to environmental concerns regarding release of products containing genes that confer resistance to drugs (such as the *E.coli* gpt gene), we have since redesigned our transfer vector plasmid to enable a second round of recombination after removal of selection pressure (called transient dominant selection) to remove the markers, resulting in a marker-free recombinant. This was performed in our laboratory for a new recombinant vaccine construct expressing the protective GPs of RVFV, called LSD-RVF.mf, as described in this paper.

This study demonstrates that the LSD-RVF.mf vaccine was safe in cattle. It was not detected in the blood following vaccination, indicating that the vaccine is attenuated, unlike virulent LSDV, which can be detected in blood following infection. The LSD-RVF.mf vaccine elicited protective immunity against both LSDV and RVFV challenge. It is likely that both antibody (humoral) and cell-mediated immunity elicited by the LSD-RVF.mf vaccine are important for protection against both diseases. Low levels of neutralizing antibodies specific for LSDV and RVFV were elicited by the LSD-RVF.mf vaccine prior to challenge. Protection against RVFV challenge in the vaccinated group of animals appears to be associated with neutralizing antibody development to the virus.

Cellular immunity was measured using the same parameters as described in a related study (22). ELISPOT assays were used to determine the number of IFN-γ-producing cells in calves and the results demonstrated that LSD-RVF.mf-vaccinated animals had higher numbers of IFN-γ-producing cells compared to calves in the control groups following challenge with LSDV and stimulation with LSDV antigen. The increased number of IFN-γ-producing cells correlated with increased CD4+ and CD8+ memory T cells in PBMCs of the vaccinated animals post-challenge. Production of IFN-γ by memory T cells is a key element required for the control of virus infections (33). It was also demonstrated that the PBMCs of vaccinated calves produced IFN-γ 7 dpi when stimulated with RVFV antigen and a RVFV-specific memory CD8+ T cell response was detected at 14 dpi. The production of IFN-γ may be considered as a key factor for survival in RVFV infection. It has been shown that IFN-γ treatment can reduce RVF infection in rhesus monkeys (34) and in other monkey models early production of IFN-γ and CD8+ T cells were detected in survivors (35). The source of IFN-γ at 7 dpi is most likely NK cells (36), but CD8+ T cells cannot be ruled out, since elevated levels of both were detected in the PBMCs of calf C2-4 at 7 dpi.

This study demonstrates that the currently used commercial OBP LSD vaccine can be used as a vaccine vector in cattle for RVFV protective antigens. This vaccine has DIVA capability for RVF enabling improved surveillance of RVF. This vaccine also has the potential to improve vaccination coverage for RVF, as the current vaccines are not used consistently, due mainly to cost factors. Further work is required for registration of this vaccine to allow it to be used in the field, although it has undergone small-scale batch formulation and the process parameters at a small scale have been determined by Onderstepoort Biological Products (OBP Ltd) in South Africa. The vaccine is safe to use and protects cattle against LSD and RVF, as demonstrated in this pilot phase cattle trial. However, recent updates in regulatory requirements for the biological license application for new veterinary vaccines necessitates additional regulatory process undertakings toward product registration for a marketing license in terms of the Medicines and Related Substances Act, 1965 (Act No. 101 of 1965), as gazetted by the government of South Africa. This will require vaccine pilot batch production, including master seed characterization and clinical validation of this vaccine batch in cattle for safety and efficacy. Plans are in progress to enable these additional studies.

## Data Availability Statement

The datasets generated for this study are available on request to the corresponding author.

## Ethics Statement

The animal study was reviewed and approved by ARC-OVI Animal Ethics Committee.

## Author Contributions

DW constructed the insertion vector, generated and selected the recombinant virus construct to homogeneity (with assistance of Mr. Maanda Phosiwa), was directly involved in most of the cattle trial (and, data analysis) and co-wrote this manuscript. AM and PK co-conceptualized, planned and executed the trial, and analyzed data and writing, review and editing of the original draft manuscript. AP planned and helped analyse the CMI work, as did NT and TN, both of whom also performed the work. AP, NT, and TN contributed to writing the manuscript. SB assisted with the cattle trial planning and helped analyse the resulting data and co-wrote the manuscript. LN and NM designed and executed vaccine formulation experiments, manufactured vaccine, and challenge strains used in the cattle trial. NM contributed to the design of the cattle trial.

## Conflict of Interest

The authors declare that the research was conducted in the absence of any commercial or financial relationships that could be construed as a potential conflict of interest.
