# Supplementary figures and images for: Protection of Cattle Elicited Using a Bivalent Lumpy Skin Disease Virus-Vectored Recombinant Rift Valley Fever Vaccine
Source: Front Vet Sci. 2020 May 19;7:256. doi: 10.3389/fvets.2020.00256 (PMC7248559; doi:10.3389/fvets.2020.00256)

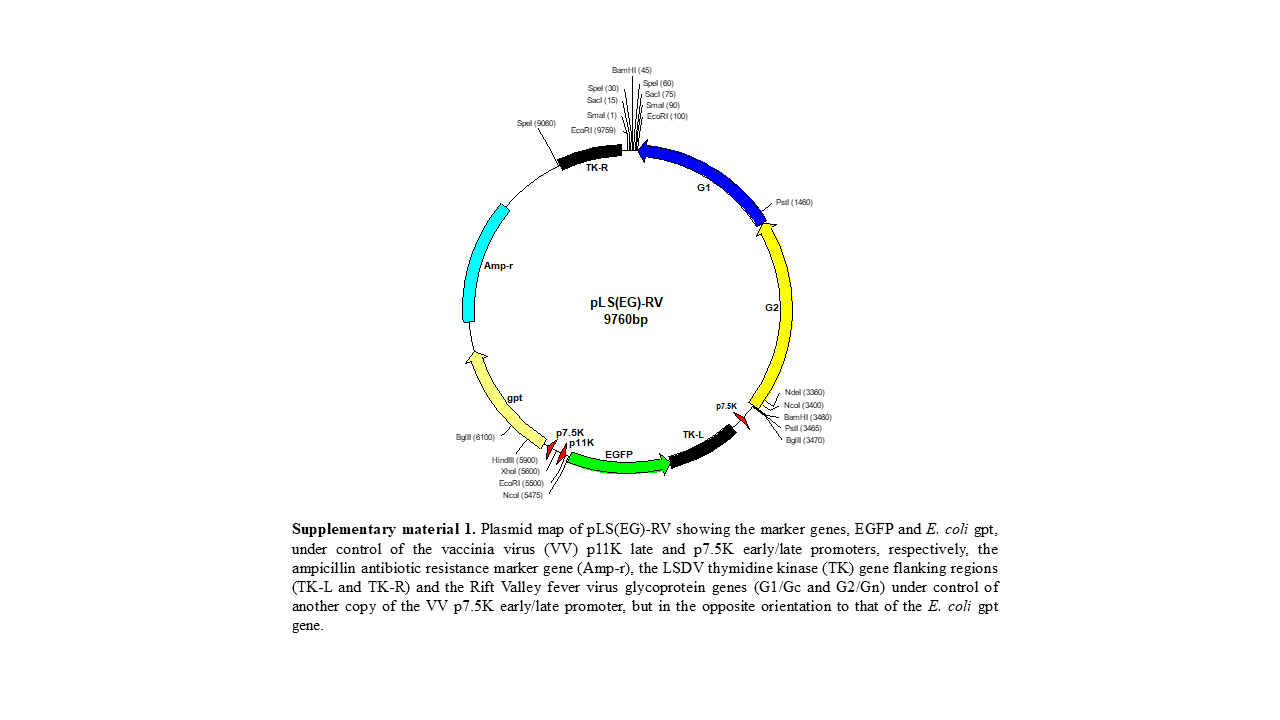

Supplement: Supplementary file 1 [file Image_1.tif]

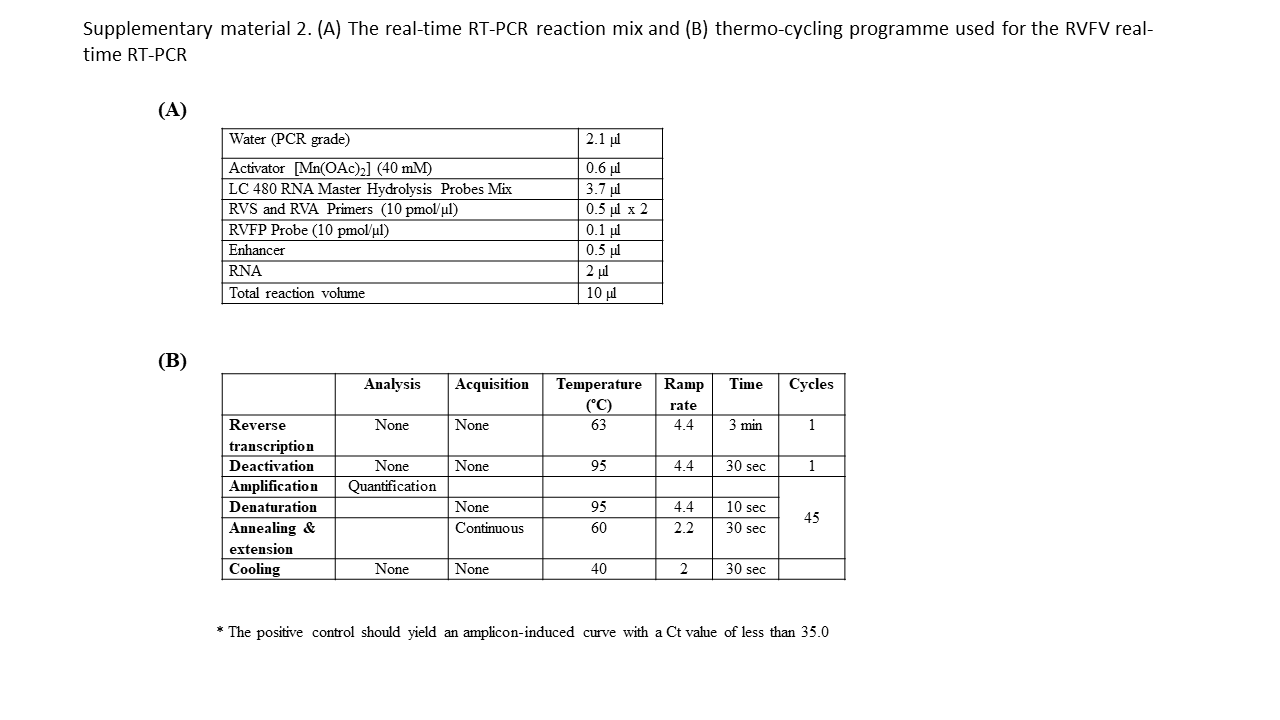

Supplement: Supplementary file 2 [file Image_2.tif]

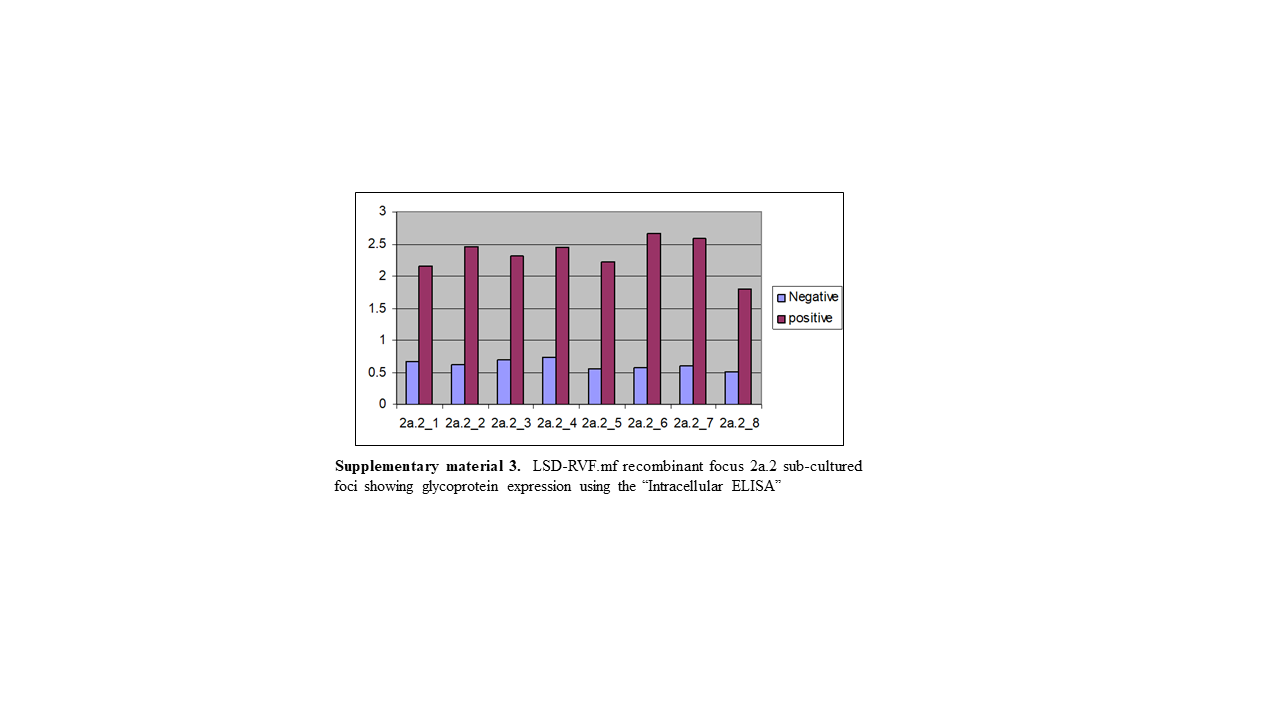

Supplement: Supplementary file 3 [file Image_3.tif]

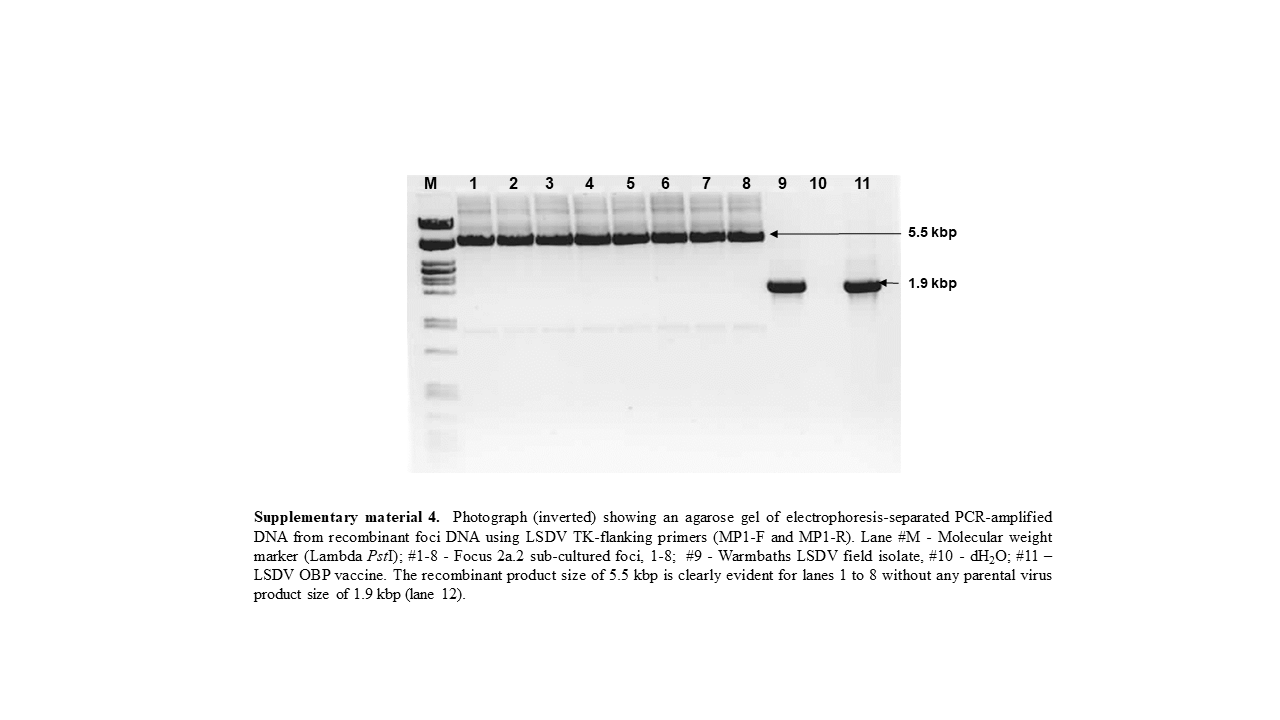

Supplement: Supplementary file 4 [file Image_4.tif]

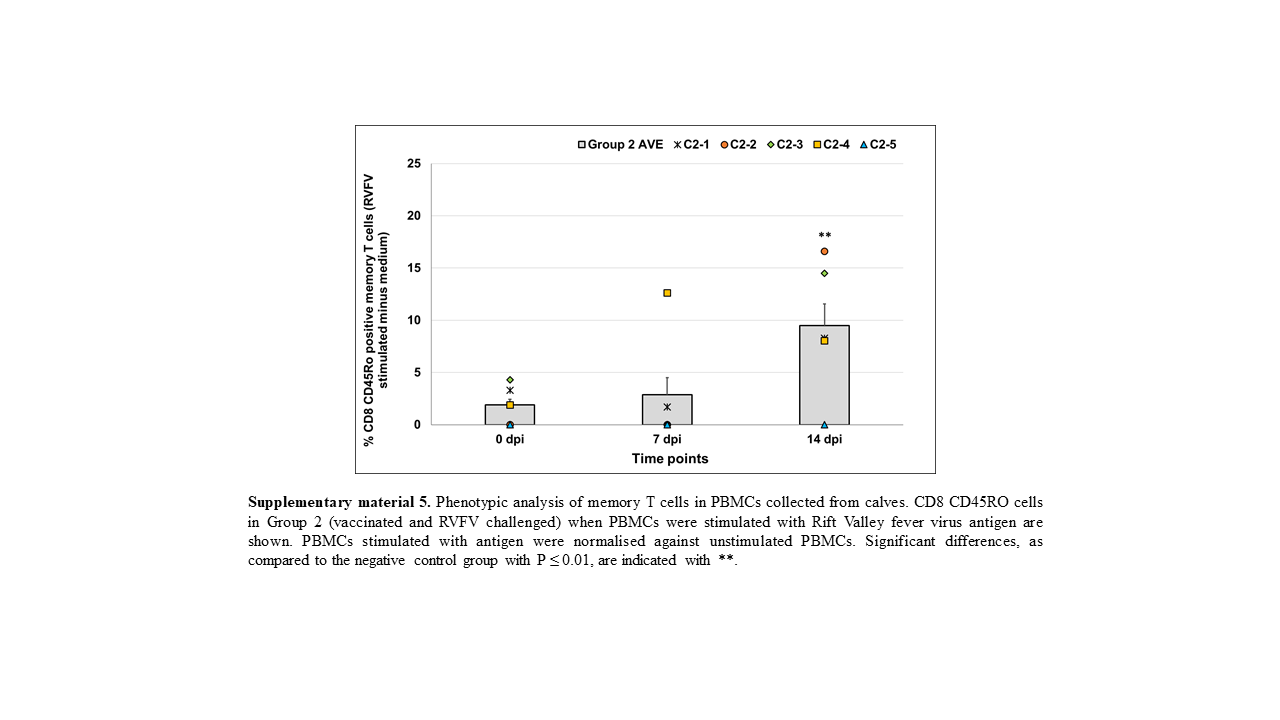

Supplement: Supplementary file 5 [file Image_5.tif]
